# Association between Single Nucleotide Polymorphisms of *PRKD1* and *KCNQ3* Gene and Milk Quality Traits in Gannan Yak (*Bos grunniens*)

**DOI:** 10.3390/foods13050781

**Published:** 2024-03-02

**Authors:** Xiaoyong Ma, Guowu Yang, Juanxiang Zhang, Rong Ma, Jinwei Shen, Fen Feng, Daoning Yu, Chun Huang, Xiaoming Ma, Yongfu La, Xiaoyun Wu, Xian Guo, Min Chu, Ping Yan, Chunnian Liang

**Affiliations:** 1 Key Laboratory of Animal Genetics and Breeding on Tibetan Plateau, Ministry of Agriculture and Rural Affairs, Lanzhou 730050, China; abdullah33@163.com (X.M.); xueshengyangguowu@163.com (G.Y.); juanxiangzhang@163.com (J.Z.); marong202017@163.com (R.M.); shenjw9090@163.com (J.S.); feng990111@163.com (F.F.); ydn9907@163.com (D.Y.); johnchun825@163.com (C.H.); maxiaoming@caas.cn (X.M.); layongfu@caas.cn (Y.L.); wuxiaoyun@caas.cn (X.W.); guoxian@caas.cn (X.G.); chumin@caas.cn (M.C.); pingyanlz@163.com (P.Y.); 2 Key Laboratory of Yak Breeding of Gansu Province, Lanzhou Institute of Husbandry and Pharmaceutical Sciences, Chinese Academy of Agricultural Sciences, Lanzhou 730050, China; 3 Institute of Western Agriculture, The Chinese Academy of Agricultural Sciences, Changji 831100, China

**Keywords:** milk quality, *PRKD1* gene, *KCNQ3* gene, single nucleotide polymorphism, Gannan Yak

## Abstract

*Protein kinase D1 (PRKD1)* functions primarily in normal mammary cells, and the potassium voltage-gated channel subfamily Q member 3 (*KCNQ3*) gene plays an important role in controlling membrane potential and neuronal excitability, it has been found that this particular gene is linked to the percentage of milk fat in dairy cows. The purpose of this study was to investigate the relationship between nucleotide polymorphisms (SNPs) of *PRKD1* and *KCNQ3* genes and the milk quality of Gannan yak and to find molecular marker sites that may be used for milk quality breeding of Gannan yak. Three new SNPs were detected in the *PRKD1* (g.283,619T>C, g.283,659C>A) and *KCNQ3* gene (g.133,741T>C) of 172 Gannan lactating female yaks by Illumina yak *c*GPS 7K liquid-phase microarray technology. Milk composition was analyzed using a MilkoScan^TM^ milk composition analyzer. We found that the mutations of these three loci significantly improved the lactose, milk fat, casein, protein, non-fat milk solid (SNF) content and acidity of Gannan yaks. The lactose content of the TC heterozygous genotype population at g.283,619T>C locus was significantly higher than that of the TT wild-type population (*p* < 0.05); the milk fat content of the CA heterozygous genotype population at g.283,659C>A locus was significantly higher than that of the CC wild-type and AA mutant populations (*p* < 0.05); the casein, protein and acidity of the CC mutant and TC heterozygous groups at the g.133,741T>C locus were significantly higher than those of the wild type (*p* < 0.05), and the SNF of the TC heterozygous group was significantly higher than that of the mutant group (*p* < 0.05). The results showed that *PRKD1* and *KCNQ3* genes could be used as candidate genes affecting the milk traits of Gannan yak.

## 1. Introduction

Yak is an important species in the Qinghai–Tibet Plateau (QTP). The means of production such as meat, milk and fur produced by yak are closely related to the life and economic income of herdsmen in the Qinghai–Tibet Plateau area [1]. Among them, yak milk is a nutritious and widely consumed food, known as “liquid gold” [2]. Compared with cow milk, yak milk is rich in all kinds of nutrients (except phosphorus) [3], cholesterol and sphingomyelin content is also higher [4]. In the Qinghai–Tibet region, people cannot often eat vegetables under plateau climate conditions; interestingly, people there do not have related nutritional deficiencies. It has been found that yak milk not only supplements most of the energy consumed by people’s daily activities, but also provides vitamins and minerals needed by the human body [5,6]. Yak milk contains more protein, casein, and fat than European dairy cows (*Bos taurus*) [7]. In China, tens of thousands of tons of yak milk and its dairy products are produced every year [8]. Gannan yaks are grazed in natural pastures all year round without using additives and antibiotic drugs [9]. Therefore, Yak milk has great potential for development and utilization, and is an ideal raw material for producing various dairy products such as butter, ghee, cheese, yogurt, etc. Among them, ghee is widely used in the religious activities of Tibetan Buddhism, and the amount of use is huge. At present, lactating female yaks grazing in natural pastures still have the disadvantage of low milk yield, with an average milk yield of about 3.18 Kg/d [10]. A lot of research is needed to improve milk yield and milk quality [11].

The *PRKD1* gene encodes protein kinase D1 (*PKD1*) [12]. The protein kinase D (*PKD*) family of serine/threonine protein kinases has three members: *PKD1-3* plays a role in diacylglycerol and related signaling pathways. *PKDs* are involved in cell proliferation, migration, differentiation, angiogenesis and immune response [13]. *PKD1* prevents epithelial-mesenchymal transition (EMT) and maintains epithelial phenotype [14]. In addition, *PKD1* also inhibits directional cell migration by blocking the actin recombination process at the forefront of migrating cells [15,16]. These results indicate that PKD1 is a key protein that inhibits the invasive phenotype in breast cancer, and the expression regulation ability of *PKD1* is down-regulated in breast invasive ductal carcinoma [17], mammary gland health is the key to ensure milk yield and milk quality of dairy cows [18]. At present, a large number of *PRKD1* gene related studies mainly focus on breast cancer. In the yak, *PRKD1* gene is located on chromosome 17, and the correlation between *PRKD1* gene polymorphism and milk quality in yak has not been mentioned. Previously, we found that three SNPS in the *CCSER1* gene were associated with milk quality (milk fat, protein, and casein) in Gannan yaks [19]. Regions reported to affect milk quality traits in dairy cows are concentrated on chromosomes 1, 6, 11, 13, 14, and 18 [20]. The *KCNQ3* gene is located on chromosome 18 of yak. According to the results of Zhou et al. [20], we speculated that the gene may be a candidate gene affecting the milk quality traits of yak. In addition, Kolbehdari et al. [21] found a SNP (rs41580517) related to milk fat content in the intron of the potassium voltage-gated channel subfamily *KQT3*-*KCNQ3* gene located in bovine BTA14. *KCNQ* channels in the central nervous system neurons, the main subunits are *KCNQ2*, *3* and *5* [22], these genes are regulated by various signaling pathways, and they play a crucial role in controlling membrane potential and neuronal excitability [23,24]. These channels produce muscarinic-inhibited potassium currents that control excitability, known as M currents [25]. The information RNA of *KCNQ3* gene exists in the brain, including cerebral cortex, cerebellum, basal ganglia and hippocampus. At present, the research on *KCNQ3* gene mainly focuses on diseases such as epilepsy, neuropathic pain and anxiety disorders [26].

Yak milk has a high concentration of nutrients and has a good therapeutic effect on some diseases [27]. Studies have shown that the milk of multiparous yaks contains more unsaturated fatty acids than that of primiparous yaks [28]. In addition, yak milk also contains other important nutritional values [29,30]. Yak milk contains several important trace elements, such as zinc, iron, copper, manganese and selenium. Zinc plays an important role in immune function, wound healing, cell growth and division [31]. Iron is an important element to produce hemoglobin, and hemoglobin is the carrier of oxygen in animals, which is crucial in the hypoxic plateau environment. It is reported that yak milk can prolong the survival time of mice under normal hypoxic conditions and improve their red blood cells and hemoglobin levels; thus, yak milk may indirectly improve the oxygen-carrying function of plateau people [32,33]. As an important antioxidant, selenium helps to protect cells from damage [32]. In the Qinghai–Tibet Plateau region, people can withstand the environment of strong ultraviolet light. On the one hand, they have adapted to this environment over a long period of evolution. On the other hand, it may be that the selenium contained in yak milk enhances the skin’s ability to resist ultraviolet light [34]. In recent years, there has been increasing interest in developing the potential of yak milk [35]. However, due to geographical location and folk culture, yak milk has not been fully developed and utilized. Herders are still mainly manual milking, and the work efficiency is low, in addition, the disadvantage of low milk yield of yak has not been improved. At present, there are few studies on the relationship between milk yield and milk quality in yaks, and there is no study on the relationship between *PRKD1* and *KCNQ3* gene polymorphisms and milk quality in yaks. The purpose of this study was to investigate the genetic polymorphisms of *PRKD1* and *KCNQ3* genes and their relationship with milk quality in Gannan yak, and to provide a scientific basis for improving milk quality and marker-assisted selection (MAS) in yak breeds (population).

## 2. Materials and Methods

### 2.1. Ethics Approval

The Lanzhou Institute of Husbandry approved all the animal experiments as well as the Pharmaceutical Sciences of the Chinese Academy of Agricultural Sciences (CAAS) with the grant number: No. 1610322020018.

### 2.2. Experimental Animals and Sample Analysis

This experiment was conducted in July 2023. The experimental animals were selected from the same natural pasture in Gannan Tibetan Autonomous Prefecture, with an average altitude of 3000 m. Milk samples were collected from 172 lactating yaks with 2 or 3 fetuses. The milk’s composition was analyzed using a MilkoScan^TM^ milk composition analyzer (Danish FUCHS Analytical Instruments Co., Ltd., Hellerup, Denmark). The measured indicators included milk fat, lactoprotein, lactose, casein, non-fat milk solid (SNF) content, acidity and total solid (TS) content.

### 2.3. DNA Samples

The ear tissues of 172 Gannan yaks were collected using U-shaped ear forceps. The collected tissues were placed in a 1.5 mL frozen tube and stored in liquid nitrogen. After being brought back to the laboratory, the animal tissue genomic DNA was extracted using the magnetic bead method (DP341, Tiangen Biochemical Technology Co., Ltd., Beijing, China) [36]. For specific extraction steps, please refer to the kit instructions used in this assay. After extraction, the concentration of DNA samples was detected by quantum bit fluorescence quantitative analyzer. The integrity of DNA samples was checked by electrophoresis on a 1% agarose gel.

### 2.4. Genotyping

Genotyping was performed on 172 Gannan yaks using the Illumina Huazhi Biotechnology Co., Ltd. (Changsha, China). *c*GPS 7K liquid chip. The genotyping was carried out by precisely sequencing liquid capture targets. The *c*GPS applies an optimized thermodynamic stability algorithm model to create a unique probe for a specific interval sequence. The synthesized probes are utilized to capture and enrich multiple target sequences located at different genomic locations via liquid-phase hybridization. The target intervals were captured and enriched, followed by library construction and next-generation sequencing to obtain the genotypes of all SNP/InDel marker loci in the target interval. After identifying the low-quality reads, we screened and removed them. If the ratio of bases with a quality value (Q) of 20 or lower in the reads was over 50% of the total bases, then we deleted those reads. Additionally, we filtered out reads with too many N bases. Only reads with less than or equal to 5 N bases were kept, and we removed reads that were less than 100 bases long. The genomic location of this SNP was determined using the assembly of the yak reference genome Bosgru v3.0 [37] (GCA_005887515.1).

### 2.5. Statistical Analysis

I used the online software GDICALL (http://www.msrcall.com/GDICALL.aspx, accessed on 7 October 2023) to calculate the homozygosity (HO) of our sample. We use Cervus 3.0 (https://www.softpedia.com/get/Science-CAD/Cervus.shtml accessed on 7 October 2023) to calculate two loci heterozygosity (HE), the effective number of alleles (NE) and polymorphism (PIC), genotype and allele frequency. I also calculated *p* values for the chi-square test and the Hardy–Weinberg test.

We used one-way analysis of variance (ANOVA) of IBM SPSS Statistics 25 (IBM, Armonk, NY, USA) to explore the relationship between *PRKD1* and *KCNQ3* gene polymorphisms and milk production traits in yaks. To analyze the factors that influence yak milk production traits, a general linear model was used and simplified based on the actual situation. Equation (1) shows the simplified model that was used. Here, *Yi* represents the phenotypic value of milk traits, *μ* is the population mean of milk fat traits, *SNPi* is the fixed effect of genotype category at this locus, and *e* is the random error effect. Duncan’s multiple comparison test was carried out to determine the difference between the mean values. The results were expressed as mean ± standard deviation. *p* < 0.05 was considered significant.

*Yi* = *µ* + *SNPi* + *e*. (1)

## 3. Results

### 3.1. Conducted Genotyping Analysis and Examined Genetic Parameters of PRKD1 and KCNQ3 in Gannan Yak

The genotyping analysis of Gannan yak showed that there were T>C substitution (g.283,619T>C) and C>A substitution (g.283,659C>A) in the *PRKD1* gene on chromosome 17, and T>C substitution (g.133,741T>C) in the *KCNQ3* gene on chromosome 18, which were located at 43,090,728, 43,090,768 and 11,881,240, respectively. As presented in Table 1, the 3 SNP loci showed 3 genotypes in the Gannan yak population. The genotypes of three SNP sites, *PRKD1* gene g.283,619T>C, g.283,659C>A, and *KCNQ3* gene g.133,741T>C, were analyzed. The most frequent genotypes observed were TC, CA, and TC; these genotypes had frequencies of 0.512, 0.494, and 0.475, respectively, indicating that the heterozygous type was dominant in all three SNP sites. In the genetic sequence g.283,619T>C, the frequency of the T allele was 0.349, while the frequency of the C allele was 0.651. This suggests that the mutant allele is predominant at this locus. In the genetic sequence g.283,659C>A, the frequency of the unmutated C allele was 0.605, implying that there are a large number of unmutated alleles. In the genetic sequence g.133,741T>C, C allele frequency was higher than T, indicating more mutant alleles. According to the genetic diversity classification of PIC (PIC < 0.25, low polymorphism; 0.25 < PIC < 0.5, moderate polymorphism; PIC > 0.5, high polymorphism) analysis found that the PIC values of g.283,619 T>C, g.283,659 C>A and g.133,741 T>C were 0.351, 0.346, 0.362, respectively, which were moderate polymorphisms. The genotype frequencies of these three loci in the Gannan yak population conformed to the Hardy–Weinberg equilibrium (*p* > 0.05).

### 3.2. Association Analysis of Milk Traits and Genotypes of SNPs in Gannan Yak

The association analysis of single nucleotide polymorphisms (SNPs) genotypes of *PRKD1* and *KCNQ3* genes with yak milk composition in Gannan yak was shown in Table 2. The results showed that the g.283,619T>C locus was significantly correlated with the lactose traits of Gannan yak milk, and the lactose content of TC type yak population was significantly higher than that of TT type and CC type (*p* < 0.05), indicating that the mutation of *PRKD1* gene improved the milk traits of Gannan yak; there were no significant differences in casein, protein, milk fat, non-fat milk solids (SNF), acidity and total milk solids (TS) among the three genotypes of yak milk (*p* > 0.05). The g.283,659 C > A locus was significantly correlated with milk fat content; the milk fat content of CA heterozygous genotype animal population was significantly higher than that of CC and AA genotypes (*p* < 0.05). The correlation analysis between g.133,741T>C locus and milk composition showed that the casein, protein and acidity of CC and TC groups were significantly higher than those of TT group (*p* < 0.05). The SNF of heterozygote TC group was significantly higher than that of CC group (*p* < 0.05). The results showed that the site mutations of *PRKD1* (g.283,619T>C, g.283,659C>A) gene and *KCNQ3* (g.133,741T>C) gene increased the casein, protein, milk fat and lactose traits of Gannan yak milk, and improved the milk quality of Gannan yak.

## 4. Discussion

Yak milk is a naturally concentrated milk that is softer and sweeter than regular milk [38]. Yak milk protein contains 80% casein, which is much higher than human milk [39]. These casein proteins consist of a group of phosphoproteins that form a gel structure during the coagulation process, effectively improving the texture and nutritional value of dairy products such as cheese and yogurt, which is the main reason why yak milk is whiter in color than cow milk [32]. In this study, the mutation of g.133,741T>C locus had a significant effect on the content of casein and protein in Gannan yak, the content of casein and protein in CC mutant and TC heterozygous yak populations was significantly higher than that in TT wild type, which indicated that the SNPs mutation of *KCNQ3* gene had a significant effect on the improvement of yak milk quality. Studies have shown that the protein of yak milk is divided into four caseins (α_S1_-CN, α_S2_-CN, β-CN, k-CN) and five major whey proteins (α-lactalbumin, β-lactoglobulin, lactoferrin, serum albumin and immunoglobulin) [40,41]. As a dietary protein, casein is a source of antihypertensive peptides, which is positive for conditioning the body of patients with cardiovascular disease [42]. It has been reported that whey protein can be rapidly digested in the digestive tract and can quickly supplement amino acids for the body [43]. Previously, researchers used iTRAQ-labelling proteomics to find 183 proteins in the whey of yak milk and colostrum, of which 86 proteins were significantly different between colostrum and milk [44]. Bioactive peptides found in yak milk play crucial roles in the metabolic and overall health of humans. They perform various physiological functions and can enhance the resistance of neonates and adults against diseases and pathogens [45]. We found that the mutations of g.283,619T>C and g.283659C>A had no significant effect on casein and protein of Gannan yak. It is worth noting that the mutation of g.283,659C>A site had an effect on milk fat, and the milk fat content of CA heterozygous type was significantly higher than that of CC wild type and AA mutant type. Yak milk has a high milk fat content and large fat globules, so it can produce high-quality ghee products [4]. Yak milk is rich in monounsaturated fatty acids (MUFAs, 20–25%) and polyunsaturated fatty acids (PUFAs, 3–6%), which play an important role in reducing the risk of cardiovascular disease and improving lipid metabolism [46]. Since yak graze in natural pastures all year round, this means yak milk may contain unique fatty acids [47]. Our results showed that the TC heterozygote at the g.133,741T>C locus had significantly higher non-fat milk solid than the CC mutant, and the acidity of the TC and CC genotypes was significantly higher than that of the TT wild type.

Advances in molecular biotechnology have enabled the detection of the contribution of some genes to genetic variation in important economic quantitative traits [21]. Georges et al. first performed a genome-wide scan of dairy cows to determine the genomic region of the genes responsible for phenotypic variation in production traits in dairy cows [48,49]. It can be seen from the quantitative trait loci (QTL) that there are many loci affecting the quantitative traits of dairy cows. At present, single nucleotide polymorphism (SNP) has been used for QTL detection and mapping of complex traits in many species [50]. In our study, when analyzing the correlation between *PRKD1* and *KCNQ3* gene loci and milk traits, it was found that when the *PRKD1* g.283,619 T>C locus was mutated from T to C, the heterozygote of the mutation site had a significant effect on the lactose of Gannan yak, and the heterozygote was significantly higher than the wild type. The heterozygote genotype composed of *PRKD1* g.283659 C>A mutation site had a positive effect on milk fat. There was no significant difference in casein, lactoprotein, SNF, acidity and TS between the *PRKD1* mutant population and the wild type. When the base of *KCNQ3* g.133,741T>C mutation site changed from T to C, it had a positive effect on the content of casein, lactoprotein, SNF and acidity of Gannan yak. In a word, the SNPs mutation sites we found have different effects on the milk quality of Gannan yak.

As a serin/threonine kinase expressed in normal breast cells, the *PRKD1* gene has been proved to be a key protein to inhibit the invasive phenotype in breast cancer [17]. In our study, we also confirmed the significant effect of *PRKD1* gene on milk quality traits of yak. In addition, *KCNQ3* has been identified as a candidate gene that determines the association of SNP markers (rs41580517) with milk fat content [21]. This study also found that the *KCNQ3* g.133,741T>C locus was associated with several milk components in Gannan yak. Therefore, our findings provide candidate genes for improving milk quality traits of Gannan yak, and also lay a foundation for subsequent research on improving milk yield of yak through screening. It is worth noting that the three loci g.283,619T>C, g.283659C>A and g.133,741T>C were located on the intron, but this does not exclude its effect on yak milk quality. The intronic regions of genes cannot be encoded and expressed, but they also play an important role in the regulation of gene expression [51]. It has been reported that the intron SNPs of *SLC22A4* gene affect the transcription efficiency of the gene [52], which strongly indicates the important role of introns in the gene. In previous studies, five SNPs in the intron region of *SORBS1* gene were significantly associated with milk fat traits [53]. This is similar to the results of our study. In addition, the polymorphism information content of the three loci g.283,619T>C, g.283659C>A and g.133,741T>C were 0.351, 0.364 and 0.362, respectively, and the *p* values of the three loci were 0.104, 0.673 and 0.970, respectively, all of which were greater than 0.05, indicating that each locus was in the Hardy–Weinberg equilibrium. Therefore, PRKD1 and KCNQ3 genes can be used as DNA molecular markers to improve milk production traits in yaks using marker-assisted selection.

## 5. Conclusions

This study is the first to investigate the association between *PRKD1* and *KCNQ3* gene polymorphisms and dairy traits in Gannan yaks. The results of association analysis showed that the polymorphisms of *PRKD1* gene and *KCNQ3* gene were significantly related to the milk quality of yaks. Therefore, genotyping *PRKD1* and *KCNQ3* genes is helpful to improve the milk quality of Gannan yaks.

## Figures and Tables

**Table 1:** Information regarding the variation and diversity parameters of the *PRKD1* and *KCNQ3* loci.

SNPs	Genotypic Frequencies			Allelic Frequencies		He	Ne	PIC	*x* ^2^	*p* Value
g.283,619T>C	TT (15)	TC (83)	CC (64)	T (113)	C (211)	0.454	1.833	0.351	2.649	0.104
	0.093	0.512	0.395	0.349	0.651					
g.283,659C>A	CC (58)	CA (80)	AA (24)	C (196)	A (128)	0.478	1.916	0.364	0.178	0.673
	0.358	0.494	0.148	0.605	0.395					
g.133,741T>C	TT (24)	TC (77)	CC (61)	T (125)	C (199)	0.474	1.901	0.362	0.001	0.970
	0.148	0.475	0.377	0.386	0.614					

Note: He: heterozygosity; Ne: effective number of alleles; PIC: polymorphism. PIC < 0.25, low polymorphism; 0.25 < PIC < 0.5, moderate polymorphism; PIC > 0.5, high polymorphism; *p* > 0.05 suggests that the population gene is in the Hardy–Weinberg balance and the sample comes from the same Mendel population.

**Table 2:** Association analysis between *PRKD1* gene g.283,619T>C, g.283,659C>A loci, *KCNQ3* gene g.133,741T>C loci and milk traits of Gannan yak.

**SNPs g.283,619T>C**							
Genotype	Casein/%	Protein/%	Fat/%	SNF/%	Lactose/%	Acidity/°T	TS/%
TT	4.14 ± 0.22	4.92 ± 0.33	6.77 ± 1.80	11.13 ± 0.55	4.90 ± 0.11 b	12.46 ± 1.02	17.96 ± 2.05
TC	4.09 ± 0.32	4.89 ± 0.43	7.10 ± 2.25	11.28 ± 0.49	5.00 ± 0.16 a	12.28 ± 1.39	16.77 ± 2.62
CC	4.08 ± 0.0.27	4.90 ± 0.37	6.80 ± 2.34	11.26 ± 0.47	4.98 ± 0.15 ab	12.39 ± 1.29	16.33 ± 2.59
*p* Value	*p* = 0.777	*p* = 0.958	*p* = 0.538	*p* = 0.494	*p* = 0.024	*p* = 0.516	*p* = 0.082
**SNPs g.283,659C>A**							
Genotype	Casein/%	Protein/%	Fat/%	SNF/%	Lactose/%	Acidity/°T	TS/%
CC	4.12 ± 0.25	4.89 ± 0.35	4.93 ± 0.95 b	11.21 ± 0.50	4.98 ± 0.15	12.23 ± 1.34	16.61 ± 1.75
CA	4.0 ± 0.33	4.89 ± 0.45	5.61 ± 1.64 a	11.27 ± 0.48	4.99 ± 0.17	12.41 ± 1.31	16.43 ± 2.58
AA	4.10 ± 0.25	4.96 ± 0.33	4.84 ± 1.38 b	11.37 ± 0.37	4.97 ± 0.16	12.70 ± 1.10	15.80 ± 1.99
*p* Value	*p* = 0.595	*p* = 0.685	*p* = 0.035	*p* = 0.378	*p* = 0.816	*p* = 0.875	*p* = 0.345
**SNPs g.133,741T>C**							
Genotype	Casein/%	Protein/%	Fat/%	SNF/%	Lactose/%	Acidity/°T	TS/%
TT	3.95 ± 0.32 b	4.61 ± 0.43 b	5.50 ± 3.00	11.02 ± 0.50 ab	5.04 ± 0.12	11.47 ± 1.30 b	16.41 ± 2.90
TC	4.14 ± 0.25 a	5.00 ± 0.34 a	5.30 ± 2.62	11.38 ± 0.43 a	5.05 ± 0.11	12.68 ± 1.14 a	16.54 ± 2.55
CC	4.12 ± 0.27 a	4.89 ± 0.39 a	5.92 ± 2.54	11.22 ± 0.48 b	4.98 ± 0.14	12.39 ± 1.32 a	17.03 ± 2.52
*p* Value	*p* = 0.011	*p* = 0.000	*p* = 0.396	*p* = 0.003	*p* = 0.006	*p* = 0.000	*p* = 0.453

Note: In the same column of data, the difference between different lowercase letters was statistically significant (*p* < 0.05). The data were expressed as mean ± standard deviation.

## Data Availability

The original contributions presented in the study are included in the article; further inquiries can be directed to the corresponding author.
